# Preclinical update on regulation of intracranial pressure in relation to idiopathic intracranial hypertension

**DOI:** 10.1186/s12987-019-0155-4

**Published:** 2019-11-26

**Authors:** Sajedeh Eftekhari, Connar Stanley James Westgate, Maria Schmidt Uldall, Rigmor Hoejland Jensen

**Affiliations:** Danish Headache Center, Department of Neurology, Glostrup Research Institute, Rigshospitalet-Glostrup, University of Copenhagen, Nordstjernevej 42, 2600 Glostrup, Denmark

**Keywords:** Intracranial pressure, In vivo, Idiopathic intracranial hypertension, Choroid plexus, Cerebrospinal fluid regulation

## Abstract

**Background:**

Elevated intracranial pressure (ICP) is observed in association with a range of brain disorders. One of these challenging disorders is idiopathic intracranial hypertension (IIH), characterized by raised ICP of unknown cause with significant morbidity and limited therapeutic options. In this review, special focus is put on the preclinical research performed in order to understand the pathophysiology behind ICP regulation and IIH. This includes cerebrospinal fluid dynamics, molecular mechanisms underlying disturbances in brain fluids leading to elevated ICP, role of obesity in IIH, development of an IIH model and ICP measurements in rodents. The review also discusses existing and new drug targets for IIH that have been evaluated in vivo.

**Conclusions:**

ICP monitoring in rodents is challenging and different methods have been applied. Some of these methods are invasive, depend on use of anesthesia and only allow short-term monitoring. Long-term ICP recordings are needed to study IIH but existing methods are hampered by several limitations. As obesity is one of the most common risk factors for IIH, a rodent obese model has been developed that mimics some key aspects of IIH. The most commonly used drugs for IIH have been evaluated in vivo for their efficacy at lowering ICP in the existing animal models. These studies suggest these drugs, including acetazolamide, might have limited or no reducing effect on ICP. Two drug targets that can impact ICP in healthy rodents are topiramate and a glucagon-like peptide-1 receptor (GLP-1R) agonist. However, it remains to evaluate their effect in an IIH model with more precise and valid ICP monitoring system. Therefore, continued evaluation in the preclinical research with refined tools is of great importance to further understand the pathophysiology behind disorders with raised ICP and to explore new drug targets.

## Introduction

### Intracranial pressure and idiopathic intracranial hypertension

Elevated intracranial pressure (ICP) is observed in association with a range of brain disorders and their underlying pathophysiology is widely unknown. Actually, there is limited insight into the regulatory mechanisms of this essential balance under normal conditions and consequently also a significant lack of specific treatments. In this review, we aim to focus on the preclinical research behind ICP regulation mainly with Idiopathic Intracranial Hypertension (IIH) as a disease model. Further, we aim to discuss the existing and novel drug targets for IIH that have been evaluated in preclinical models.

The regulation of ICP is fundamental for providing a stable environment to enable normal brain function. According to the anatomists Monro and Kellie, ICP is determined by three parameters; the volume of the brain, cerebral blood volume and cerebrospinal fluid (CSF) volume, where cerebral blood volume and CSF are homeostatically controlled and compensate for acute changes in ICP [1]. Several cerebral pathologies of raised ICP exist which have profound consequences for the patients, such as: traumatic brain injury (TBI), ischemic stroke, brain tumor, hepatic encephalopathy, hydrocephalus and IIH. IIH is a rare disease with an incidence of 4.7 per 100,000 in the UK, where the disease predominantly affects females (82%) [2]. Raised ICP is an obligatory symptom of IIH, thus it is apparent that ICP homeostasis is perturbed in IIH. The diagnosis of IIH eliminates secondary causes of raised ICP, consequently altered cerebral anatomy and altered brain blood volume are unlikely to be causative in IIH. As such, CSF dynamics in IIH must be perturbed, where CSF dynamics are a balance of CSF secretion and drainage [3, 4].

### CSF secretion

**Fig. 1:**
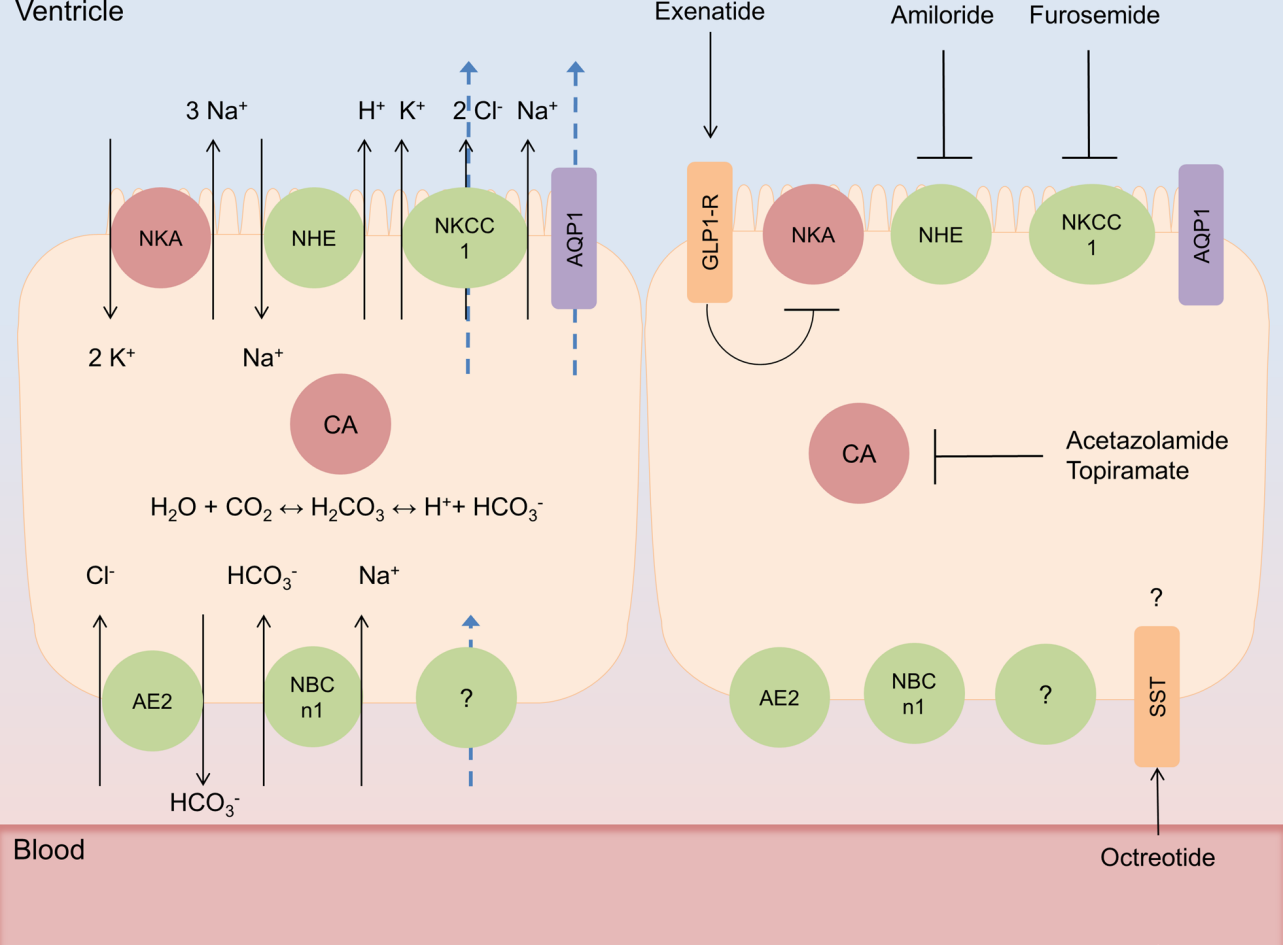
CSF secretion modulation at the choroid plexus. Simplified schematics of choroid plexus epithelial (CPe) cells. (Left cell) CSF secretion at CPe cells, whereby the activity of carbonic anhydrase (CA) generates carbonic acid which dissociates to a proton and a bicarbonate ion, these drive the sodium/hydrogen exchanger (NHE) and anion exchanger (AE2) respectively, transporting sodium and chloride ions into CPe cells. Additionally the basolateral sodium bicarbonate transporter (NBCn1) transports bicarbonate and sodium ions into the cell. These provide the ionic gradients to drive both the Na^+^/K^+^ ATPase and the NKCC1 channel to transport sodium into the ventricular spaces, this facilitates the osmosis of water via aquaporin 1 (AQP1). Furthermore the action of NKCC1 independently draws water from the cytosol to the ventricular space. The mechanisms of basolateral water transport remain unelucidated. Blue arrows represent the movement of water. CPe cell to the right represents current and proposed drug targets for modulating CSF secretion at the choroid plexus where the majority directly modifies the transport or generation of ions. The exceptions lie with somatostatin receptor (SST) agonist octreotide whose function and effect on ICP are unclear, and glucagon like peptide 1 receptor (GLP-1R) agonist exenatide which inhibits Na^+^/K^+^ATPase actity

Humans produce around 500 ml of CSF per day, filling CSF spaces to a volume of 120–150 ml, indicating an average secretion rate of around 20 ml/h. A portion of CSF is generated from cerebral interstitial fluid via the ependymal lining of the ventricles and the pia mater, likely through hydrostatic forces. However the majority of CSF is generated via the choroid plexus (CP), organs that reside in the cerebral ventricles and display the characteristic asymmetrical distribution of ion transporters of fluid transporting epithelia (Fig. 1). Although it is clear that the activity of ion transporters such as the Na^+^/K^+^ ATPase and the Na^+^-K^+^-2Cl^−^-1 cotransporter (NKCC1) are fundamental in CSF secretion at the CP, and are targeted clinically to reduce ICP (Fig. 1), the mechanisms underlying the transport of water at the CP are controversial [5, 6]. It has been hypothesized that CSF secretion at the CP is predominantly driven by osmosis, facilitated by the expression of the water channel Aquaporin 1 (AQP1). However, evidence suggests that this is not the case. The CP is relatively impermeable to water, which is likely explained by the predominantly apical expression of AQP1 on CP, preventing osmosis [7–9]. Indeed AQP1 knockout mice do not ameliorate CSF secretion [10]. Additionally the CP secretes CSF against an osmotic gradient, further demonstrating that osmosis is likely not the primary driver of CSF secretion [11]. This secretion independent of osmosis is mediated by solute-mediated water co-transport, via the NKCC1 transporter, which contributes to roughly 50% of CSF secretion in mice [6]. Although this accounts for the majority of water transport at the apical CP membrane, there is little understanding as to how water is transported across the basolateral membrane of the CP, although it is likely mediated via solute mediated water co-transport [12].

### CSF drainage

Where CSF is produced it must be drained in such a manner that is homeostatically maintained to prevent aberrant alterations in ICP. Although the mechanisms underlying the homeostasis and molecular aspects of CSF drainage have not been elucidated, the structures where CSF drainage occurs are clearly defined. The arachnoid granulations (AG) of the superior sagittal sinus are thought to drain CSF directly into the venous system [13]. The precise mechanism for CSF drainage at the AG is unknown and the contribution of AG to CSF drainage is unknown. It is hypothesized to be facilitated through either paracellular movement of water or the vacuolisation of CSF by the capsule cells of the AG [14]. However the lack of AG in rodents suggests that other modalities for CSF drainage exist.

It has been hypothesised that CSF can be drained via perivascular spaces that penetrate the parenchyma, termed glymphatics, in an Aquaporin-4 (AQP4) dependent manner [15]. This glymphatic drainage could be aided via the proposed trans-ependymal CSF flow from the ventricles to the parenchyma [16]. Additionally, two distinct branches of the lymphatic system have been demonstrated to drain CSF. The lymphatics of the nasal cavity have been demonstrated to drain CSF through the cribriform plate in a variety of mammals including humans and non-human primates [17, 18]. Additionally, dural lymphatics have the capability to drain CSF and that damage to them reduces CSF flow and drainage [19, 20].

### Regulation of ICP and IIH

There are currently a limited number of studies that assess CSF dynamics in IIH. Several studies utilizing small cohorts of IIH patients identified an increased CSF flow rate through the cerebral aqueduct, indicating increased CSF secretion [21–23]. Studies utilizing isotope cisternography suggest that IIH patients have increased resistance to CSF drainage, however these findings are not universal across IIH patients and could be confounded by increased venous sinus pressure [24–26].

Histological data from cortical biopsies of IIH patients showed increased AQP4 expression at astrocyte end feet, a constituent of the glymphatic drainage. This is suggestive of an altered state of CSF clearance via the glymphatic system [27]. Furthermore this study identifies that IIH brains have astrogliosis, perhaps explaining previous findings that IIH patients have larger ICP wave forms compared to headache controls, which indicate reduced parenchymal compliance [27, 28]. In a recent study from the same group, they demonstrated dysfunction of the blood-brain barrier (BBB) by measuring the extent of extravasated fibrinogen in IIH patients compared to reference material [29]. This study suggests that there may be leakage of BBB in IIH patients [29]. Further research is needed to evaluate the possible changes of BBB in IIH patients and what might cause the leakage.

Although it is apparent that ICP and in particular CSF dynamics are altered in IIH, the current literature fails to elucidate conclusively whether IIH patients have CSF hypersecretion, reduced CSF drainage or a combination of these factors. Furthermore the studies do not address the etiology of the alterations they describe, consequently further studies are required.

### ICP dynamics in IIH

The gold standard for measuring ICP in patients is by using an intracranial probe however, this requires neuro-surgical intervention with risk of infections. Lumbar puncture (LP) opening pressure measured during an LP is less invasive however, the obtained ICP values are less accurate than the gold standard method, and can only provide a short-term pressure reading during the procedure. There are some LP measurement methods that have been shown to be comparable to intraparenchymal ICP recordings [30–32]. The LP opening pressure is a common procedure in the diagnosis of IIH but is not always standardized. According to the latest consensus, the diagnostic criteria mandate a cut-off opening pressure of > 25 cm CSF (18.3 mmHg) for diagnosing IIH [4]. However, this absolute value for IIH diagnosis is widely discussed as most clinicians experience a ‘grey zone’ between 25 cm CSF and 30 cm CSF, which may not be pathological [33]. Further, it is also recognized in the consensus that this cut-off value is based on a single LP measurement and it should be recognized that there is a diurnal and wide variation in CSF pressure in patients [4]. It also highly debated if the LP opening pressure may not provide the true steady-state pressure because of normal fluctuations in ICP. Although mean ICP may decrease with responsive treatments, it has been shown that IIH patients still had raised mean ICP wave amplitude compared to controls, suggesting that the pulsatile ICP may be more relevant than the static ICP in the diagnostic setting for patients with IIH [28]. Another issue is that there are no true reference values for ICP in healthy humans. In one study, ICP was investigated in healthy elderly people (60–82 years) with no psychiatric or neurologic disorders and these values could be used as reference values in the elderly [34]. Otherwise, what is considered to be normal values of ICP is based on examinations in patients needing ICP monitoring due to suspected disturbance in ICP. In some clinical studies, a control group of patients considered to be “pseudonormal” have been used in order to better understand the ICP values in humans. From these studies, the effect of body posture on ICP differed between healthy and patients with conditions with elevated ICP, where in normal human physiology patients appeared to have more tightly regulated ICP when switching body posture [35, 36]. ICP reference values are currently estimated to be 7–15 mmHg in a horizontal position, however these values can be lower and negative and should be considered to be normal (supine position, mean ICP 0.5 mmHg; vertical position, mean ICP − 3.7 mmHg) [37]. Further, it has also been demonstrated that the “pseudonormal” group of patients had higher night time ICP compared to day time during 24 h ICP monitoring [38]. In addition, the authors reported decrease of ICP with age across all ages [38]. The remaining question is if these values might be similar in healthy subjects. Since ICP monitoring in healthy subjects is ethically unacceptable, a study evaluating ICP regulation with detailed waveform analysis in normal rodents would be of great value.

In rodents, ICP values have been reported to vary between 2 and 8 mmHg with a mean value around 6 mmHg [39, 40]. In animal models with raised ICP such as hydrocephalus, TBI and stroke, ICP is raised to 10–30 mmHg [39, 41, 42]. The question is to what extent ICP should be raised in an animal model of IIH. As an IIH model should not cause severe damage, we predict that mean ICP should be above 6–7 mmHg but probably not extending as high as in the severe cerebral trauma models.

## IIH pathophysiology

As mentioned earlier, IIH is a challenging disease with increased ICP of unknown aetiology and was previously called benign intracranial hypertension or pseudotumor cerebri. The most disabling symptoms are chronic headache, impaired vision due to optic nerve compression and cognitive impairment [43–45]. IIH occurs mainly in young female individuals, 3.5 in 100,000 in the general population, but the incidence is rapidly increasing in the wake of the global obesity epidemic, with up to 20 in 100,000 in the obese population [3, 46–48]. The higher incidence and increased awareness may have improved the visual prognosis, whereas disabling headaches and cognitive dysfunction persist and lifelong disability may be the result [45, 49, 50]. Recent clinical evidence suggests the involvement of hormones, androgens and glucocorticoids in IIH pathophysiology [3, 51, 52]. As many IIH patients are obese, they might also have increased risk factors for cardiovascular conditions. In one study it was shown that 56% of the IIH patients had hypertension [53]. However, control patients were not included and the hypertension might have been due to obesity. In a recent large matched cohort study, the risks of cardiovascular disorders were evaluated in women with IIH compared to controls matched on BMI and age [54]. The risk of heart failure, ischemic heart disease, stroke/transient ischemic attack (TIA), type 2 diabetes, and hypertension was shown to be increased in women with IIH (30). For hypertension, the crude incidence rate was 14.1 per 1000 person-years in women with IIH and 9.2 per 1000 person-years in those without IIH (30). The underlying mechanisms for these risk factors need to be evaluated. Interestingly, it has been suggested that hypertension may alter CP dynamics affecting CSF production and causing BBB alterations [55]. Preclinical studies have demonstrated that hypertensive rats have variations in their CSF composition that could be due to CSF dysfunction, which may lead to blood-CSF barrier disruption [56]. Furthermore, it has been shown that hypertensive rats have increased CSF secretion rate and CP blood flow [57]. Therefore, it remains to be clarified if and how hypertension may affect the CSF dynamics in IIH patients.

As the molecular regulation of CSF is not yet fully understood, there is a lack of understanding for many pathological conditions associated with abnormal CSF dynamics. Hence, diseases involving elevated ICP are generally poorly understood and managed, due to the lack of specific targeted treatment options. Medical interventions for IIH range from dietary therapy (weight loss) to diuretics and surgical CSF diversion [3]. In IIH patients with rapidly declining vision, CSF diversion surgery may be necessary. Thereby, the majority of the patients receive medications that may reduce CSF secretion and consequently reduce ICP. However, all the interventions for IIH are non-specific and hampered by side effects. The 2015 Cochrane review concluded there was insufficient evidence to determine which treatments are potentially beneficial in IIH [58]. Therefore, there remains an unmet medical need for effective treatments of IIH. This highlights the need for preclinical research using animal models to increase the understating of the pathophysiology behind IIH and to explore novel drug targets.

### Role of obesity in IIH

IIH has a prominent association with both the female gender and obesity as 90% of patients with IIH are obese [3, 48, 59]. The risk of IIH also increases with increasing BMI. The greater amount of weight gain the year before symptom onset resulted in a greater risk of IIH [46]. Thereby, weight loss has been suggested as effective treatment strategy as it can lower ICP and improves some IIH symptoms [60–62]. However, typically weight loss is not maintained, meaning IIH symptoms return. Bariatric surgery has been suggested as a therapy option for IIH patients but these studies have been done in smaller patients groups without proper controls, so more studies are needed here [63, 64]. A randomized controlled trial of bariatric surgery in IIH patients has been started with the aim to compare two methods of weight loss, bariatric surgery and a dietary program, to see which offers the most effective sustainable treatment for IIH [65]. The trial is expected to be completed in 2022 (ClinicalTrials.gov, NCT02124486).

There is also a strong association between abdominal fat deposition and health outcomes consequently adipose distribution in patients with IIH may be of relevance [66]. It has been shown that preferential lower body fat accumulation occurs in IIH patients using waist-hip ratios [67]. In a more recent study the gold standard dual-energy X-ray absorptiometry (DEXA) scanning technique was used to evaluate fat mass and distribution in IIH patients. In this study the authors found instead that IIH patients have a similar centripetal distribution of fat mass to patients with simple obesity [68]. These results raise the question whether it is the type of adipose tissue or its location that is of relevance for IIH. Adipose tissue functions as an endocrine organ, secreting a myriad of factors including proinflammatory cytokines, chemokines and adipokines [66, 69]. Therefore, hormone secretion from adipose tissue may be implicated in IIH. As obesity is the most common risk factor for IIH, the impact of obesity on ICP in rodents has been evaluated by our group and is discussed below.

### Development of a rodent IIH model

Currently there are very few animal models with elevated ICP and they are associated with the induction of severe cerebral traumas such as subarachnoid hemorrhage, TBI, brain tumors, kaolin induced hydrocephalus and cerebral ischemia [39, 42, 70–73]. Development of an intracranial hypertension model without causing severe damage would be a valuable tool to increase our knowledge regarding the molecular regulation of CSF and disorders affecting the CSF production/regulation such as IIH. A model for IIH could improve the knowledge regarding the pathophysiology of this disorder as well as the molecular mechanisms behind elevated ICP in other conditions. So far only one model has been developed that mimics some aspects of IIH [74]. Since there is a strong association between obesity and IIH, a model for obesity-induced IIH has been developed [74]. Zucker rats have been used as an obesity model with a mutation in the leptin receptor gene causing hyperphagia [75, 76]. Our group has demonstrated that the Zucker rats have higher ICP compared to lean-rats, suggesting that obesity may increase ICP [74]. At the end of the study, 28 days, the Zucker rats had an average of 40% higher ICP with the tendency of the ICP to increase in parallel with increasing BMI. Interestingly, mRNA and protein expression of AQP1 in CP of the obese rats were elevated compared to lean rats however no differences in the expression of NA/K ATPase alpha 1 expression was observed [74]. Physiological factors known to potentially influence ICP such as mean arterial blood pressure (MABP), arterial blood gases, serum retinol and retinol binding protein 4 (RBP4) levels were investigated. The authors found no differences in these parameters between the obese rats and lean rats supporting that the increase in ICP was attributed to the obesity. Although the Zucker rats developed higher ICP, the question is if this a sufficient elevation to mimic the pathologically elevated ICP observed in IIH. Further, the obesity observed in IIH patients is hypothesized to be diet-induced since weight loss is associated with improvement in some of the IIH symptoms. Therefore, it will be of great interest to study ICP levels in diet-induced obese rats, which share many characteristics with the common form of human obesity. In addition, it will be of great importance for a robust IIH model to also mimic other clinically relevant IIH symptoms such as papilledema and headache. Further, researchers suggest that hormonal and metabolic factors play an essential role in the development of IIH and these co-factors also needs to be evaluated in animal models for IIH [3, 51, 66]. A recent study has also highlighted the role of androgen excess in IIH patients showing that female IIH patients had increased testosterone in both serum and CSF [52]. The in vitro data demonstrated that testosterone is able to enhance the activity of Na^+^/K^+^-ATPase at the CP, suggesting that testosterone may modulate CSF secretion [52]. It remains to evaluate the effects of testosterone in vivo to elucidate its impact on ICP and as a driver for IIH.

## ICP measurements in animal research

Another fundamental factor in the development of an IIH animal model is the ability to perform long-term ICP measurements in rodents. ICP measurements are widely used for diagnostic purposes and to inform treatment options for conditions with raised ICP such as IIH. However, the invasive nature of performing ICP monitoring in humans limits clinical research. Thereby, there is a great need of consistent and precise ICP recording in animal models, where ICP can be measured across the day, in response to a drug and during disease development. However, it has been very challenging to measure ICP accurately in animal models due to the lack of refined equipment designed specifically to measure ICP in rodents. Various locations and techniques for measuring ICP have been investigated such as epidural, subdural, ventricular cisterna magna placement using a cannula, fluid-filled catheter or fiber-optic pressure transducers [77–86], summarized in Table 1. Lumbar cannulation has also been investigated in rodents [86, 87]. The intraventricular technique in the preclinical setting is very invasive and causes severe complications such as infections and degeneration of the cerebral tissue, so only short-term monitoring is possible. Therefore, subdural and epidural cannulations are mostly used for ICP measurements in animal models. We have developed a novel method for long-term monitoring of ICP in rats from epidural space using fluid-filled catheter [40]. This study demonstrated that the epidural ICP recordings correlated exceptionally well with ventricular ICP with no complications as observed with the ventricular probes [40]. In the rats implanted with ventricular ICP probes, the probes lost patency and many of the animals developed symptoms of neurological malfunction, infections and/or displayed general signs of illness. At post mortem brain examination, hydrocephalus was observed in the rats with the ventricular probes [40]. We found that the epidural probes were less invasive with no tissue damage. These results suggest that the ventricular cannulation for ICP recordings may not be suitable for research purposes investigating molecular changes in ICP and CSF production. This study is also the longest ICP monitoring performed in rats, for up to 59 days. However, ICP recordings could not be performed daily and therefore the ICP recordings were performed on selected days with a total of 6 recordings.

**Table 1:** Overview of preclinical studies using different methods to measure ICP in rat models

Refs.	Measuring site	Measuring period	Method
[122]	Cisterna magna^a^	4 days	Fluid-filled system
[83]	Cisterna magna^a^	Unknown	Fluid-filled system
[84]	Cisterna magna^a^	1.5 h	Fluid-filled system
[123]	Cisterna magna^a^ Ventricular^a^	1 h	Fluid-filled system
[40]	Ventricular and epidural^a^	59 days	Fluid-filled system
[81]	Ventricular	Unknown	Fluid-filled system
[124]	Ventricular	28 days	Fluid-filled system
[94]	Subarachnoid space^a^	4 h	Fluid-filled system
[87]	Lumbar cannulation^a^	25 h	Fluid-filled system
[125]	Lumbar cannulation^a^	25 h	Fluid-filled system
[125]	Subdural^a^	7 days	Fluid-filled system
[77]	Epidural^a^	6 h	Fluid-filled system
[126]	Epidural^a^	1 h	Fluid-filled system
[123]	Parenchyma^a^	1 h	Fiber-optic transducer
[86]	Parenchyma^a^	14 days	Fiber-optic transducer
[127]	Parenchyma	20 h	Fiber-optic transducer
[85]	Parenchyma^a^	3 h	Fiber-optic transducer
[88]	Ventricular^a^	7 days	Telemetric device
[128]	Ventricular	2–10 days	Telemetric device
[78]	Epidural	5 days	Telemetric device
[41]	Ventricular^b^	6 days	Telemetric device
[90]	Ventricular^b^	5 days	Telemetric device
[39]	Subdural^b^	28 days	Telemetric device

^a^Indicates that the rats where anesthetized/sedated during ICP measurements

^b^Indicates continuous 24/7 ICP measurements

Although the implementation of epidural ICP recording using fluid-filled systems has been a major improvement for measuring ICP in rodents, this method has some limitations. In this method the transducer is not permanently implanted, which prevents the possibility of continuous ICP recordings over larger time intervals. Furthermore, this ICP method as the others depends on repeated use of anesthesia/sedation before each recording to immobilize the animals which are additional cofounders affecting ICP. Additionally, the fluid-filled systems can develop air bubbles, resulting in false ICP values. Fiber-optic pressure transducers have been used to avoid these problems, however, it has only been developed for animals under anesthesia [79]. Telemetry devices using fluid-filled systems were developed in order to measure small changes in ICP and with the aim to develop a system for freely moving animals. This kind of telemetry has been used in rodents however ICP monitoring in freely moving rats was limited to 2–7 days [78, 88, 89].

Recently, telemetric probes with specific ICP transducers have been developed to measure ICP continuously in freely moving rats (Kaha Sciences) [39]. This has a number of clear advantages compared to other available options in the field; it allows long-term recordings under conditions unhindered by anesthesia or restraint. So far this system has only been used in few studies, one in healthy male rats with continuous ICP monitoring for 28 days and two in a TBI model with ICP continuously monitored for 5–6 days [39, 41, 90]. Since this system does not require any use of anesthesia/sedation for ICP recording, it may be very useful to study drug effects without any impact from additional confounders. It is important to be aware that in all the in vivo drug studies discussed below, anesthesia/sedation was used in order to measure ICP and the recordings were not performed continuously.

## Drug targets for IIH

### Acetazolamide

In the management of IIH, the focus is on reducing the elevated ICP by pharmacological therapy. Given that IIH patients often suffer from visual disturbances and headache, management of IIH has been focused on preserving vision and reducing headache morbidity [4]. In this case, acetazolamide is the most commonly used drug for treatment of IIH. This drug is a carbonic anhydrase (CA) inhibitor that is believed to reduce CSF secretion and ICP via action on the CP by reducing ion transport consequently water across CP (Fig. 1). It has been suggested that acetazolamide may decrease CSF secretion by 55% in rats [91] and at similar rates in rabbits [92]. While some clinical studies have demonstrated a beneficial effect in IIH, it has limited efficacy, poor tolerability, and unresolved target mechanisms. Therefore, it is of great value to study the effect of this drug in preclinical models to understand its mechanisms and possible effects. In healthy rats, we have shown that a single high dose of acetazolamide (200 mg, given i.p.) lowered ICP and modulated the CSF secretion pathway [93]. Interestingly, we found that AQP1 and Na/K ATPase protein were increased in the membrane of CP epithelial cells, which could be a compensatory mechanism for the reduced ICP. In support, an older study also demonstrated that acetazolamide given as intravenous infusion in rats caused a reduction in ICP [94]. However, another study showed that intra-arterial injections of acetazolamide reduced ICP, whereas intraventricular injection had no effect [95]. Thus it seems that the administration route and dose of acetazolamide is important for studying its effect on ICP. In a recent study, a clinically relevant dose of acetazolamide was tested in rats via different administration routes [96]. The low dose of acetazolamide (103.3 mg/kg) that was equivalent to a single human dose (1 g) and a high dose (413.4 mg/kg) equivalent to a human daily dose (4 g) were administered either subcutaneously or orally. Neither the low nor the high dose of acetazolamide significantly lowered ICP [96]. Using the most clinically relevant delivery route for acetazolamide, oral administration, the drug lowered blood pH and induced diuresis in the rats, however it did not change ICP [96]. In this study, the authors used 4% NaCl as appropriate vehicle control as hypertonic solutions can lower ICP. This might explain the conflicting results from previous studies. In support of this study, in a rodent stroke model, acetazolamide (50 mg/kg) given i.p. improved the pathological ICP spikes but it did not impact in the mean ICP levels or the outcome [97]. However, in all these studies only a single dose of acetazolamide was examined. Therefore, it is of great importance to examine if repeated clinical doses of acetazolamide might lower ICP as IIH patients often need repeated dosing of acetazolamide. In another condition with raised ICP, treatment with acetazolamide had no effect on clinical signs or ventricular volume in dogs with internal hydrocephalus [98]. Importantly, in clinical studies, treatment with acetazolamide resulted in symptom improvements such as visual field function in IIH patients with mild visual loss [99, 100]. In the animal studies, only the direct effects on ICP were studied, as a proper IIH animal model with related symptoms is missing. Therefore, it remains to investigate the effects of acetazolamide in a pathological condition mimicking IIH in vivo. Although acetazolamide is the most common used drug for IIH, the support for its efficacy on lowering ICP is inconclusive. As many patients do not tolerate the drug due to all its severe side effects, it is even more important to further evaluate the effect of this drug and its use for IIH.

### Topiramate

Topiramate was originally used for epilepsy treatment and as a migraine prophylactic drug [101, 102]. Topiramate has many mechanisms of action via activity on four protein complexes that are regulated by protein kinase phosphorylation: voltage-gated sodium channels, high voltage-gated calcium channels, GABA receptors, and AMPA/kainate receptors [102]. In addition, topiramate is a CA inhibitor similar to acetazolamide (Fig. 1) and has therefore become of interest for IIH treatment. Topiramate is of interest for IIH headache treatment, given that topiramate is an effective migraine prophylactic and that many IIH patients suffer from severe headache. In addition, topiramate can induce favorable and welcomed weight loss which is of benefit for treating IIH patients [103]. There are some clinical case studies that have shown efficacy of topiramate for IIH symptoms [104–107]. Compared to acetazolamide, topiramate has higher carbonic anhydrase isoform specificity and increased lipophilic nature, which suggest better pharmacological activity than acetazolamide. However, in an open label study, it was shown that topiramate improved IIH symptoms but no differences were found compared to acetazolamide treatment [108]. In a recent preclinical study, it was however shown that topiramate significantly lowered ICP in healthy rats while acetazolamide did not reduce ICP [96]. Topiramate was tested at both clinical low and high doses, which lowered ICP levels by 32% and 21% respectively in healthy rats [96]. It remains to elucidate the effect of this drug on ICP in animal models with raised ICP and evaluate its effect of daily dosing as performed in the clinical studies. Although topiramate might have beneficial effects for IIH patients, this drug causes serious side effects such as cognitive impairment, insomnia, paraesthesia, fatigue and anxiety. This drug may therefore not be a better treatment option for IIH than acetazolamide.

### Amiloride and furosemide

Diuretic agents have long been known to reduce CSF secretion and have therefore been regarded as a treatment option for IIH (Fig. 1). One of these agents is furosemide, which blocks the cotransporter NKCC1 on CP epithelial cells. Furosemide has shown to reduce CSF secretion in various species [92, 109–111]. Furosemide has usually been used in combination with mannitol, where the combination has shown to reduce ICP [112, 113]. It has also been tested in combination with 3% hypertonic saline which reduced ICP in patients with ICP > 20 mmHg [114]. A rat study showed that furosemide alone did not affect plasma osmolality or brain water content at different doses, however it enhanced the effect of mannitol with greater reduction of brain water content [115]. When furosemide was given alone at high dose or low dose, this drug did not significantly reduce ICP in rats [96]. Another diuretic agent that is able to reduce CSF secretion is amiloride which inhibits the Na^+^/H^+^ exchanger and/or Na^+^ channels on the CP epithelial cells [116, 117]. One study has shown that amiloride reduced ICP in experimental brain edema in rats [118]. However, another study in healthy rats showed that amiloride and furosemide did not change ICP [96]. Clinical studies evaluating the efficacy of furosemide or amiloride for the treatment of IIH do not exist.

### Octreotide

Octreotide is a synthetic somatostatin analogue and a potent inhibitor of growth hormone. Two uncontrolled clinical studies have shown its efficacy for IIH treatment [119, 120]. In an in vivo study in rats a single dose of octreotide had no effect on ICP [96]. However, in the clinical studies, octreotide was given daily to IIH patients during the first months following diagnosis which might be necessary to significantly reduce ICP. Therefore, it will be of great interest to study repeated dosing of octreotide in animal studies.

### GLP-1R agonists

Glucagon-like peptide-1 receptor (GLP-1R) agonists are used to treat diabetes and promote weight loss. Due to its ability to affect fluid homeostasis in the kidney, we investigated if the GLP-1R agonist, exendin-4, could modulate CSF secretion and reduce ICP in rats [42]. This study demonstrated expression of GLP-1R in human and rat CP, and its ability to reduce Na^+^- and K^+^-dependent adenosine triphosphatase activity in cell cultures. Subcutaneous injections with exendin-4 showed a reduction in ICP in both healthy and hydrocephalic rats [42]. Importantly, a single subcutaneous injection of exendin-4 maintained lower ICP for 24 h. These results suggest that GLP-1R agonists may provide an alternative treatment for raised ICP conditions. It will be of great importance to investigate the effect of exendin-4 in an IIH animal model as the pathophysiology is very different from hydrocephalus. In another study it was shown that treatment with the GLP-1 analogue, Liraglutide, significantly reduced cerebral edema after experimental TBI [121]. Taken together, a clinical study investigating the effect of GLP-1R agonists on ICP in humans is highly needed.

## Conclusions

The role of obesity on ICP regulation has been evaluated in vivo and lead to the first animal model mimicking some aspects of IIH. However, a refined model is needed to further investigate the mechanism behind IIH and also to provide valuable new knowledge regarding both regulation and dysregulation of CSF and ICP dynamics. The most used drugs in the management of IIH have been evaluated in rodents and although with some limitations, some of these are not efficient for lowering ICP. However, their effect on IIH-related symptoms such as visual outcome and headache has not been evaluated in animal models. Preclinical research suggests a GLP-1R agonist as a novel drug target for elevated ICP which needs to be further evaluated. Furthermore, a novel ICP monitoring system has been developed for long-term ICP monitoring in freely moving animals, which is a prerequisite to resolve the molecular origin of IIH-elevated ICP and to evaluate drug targets. In conclusion, preclinical investigations including the development of adequate in vivo models are warranted in order to increase our understanding in IIH pathophysiology and to explore novel drug targets able to reduce ICP.

## Data Availability

Not applicable.

## Abbreviations

AG: arachnoid granulations
AQP1: aquaporin-1
AQP4: aquaporin-4
AQPs: aquaporins
BBB: blood–brain barrier
CA: carbon anhydrase
CNS: central nervous system
CSF: cerebrospinal fluid
CP: choroid plexus
CPe: CP epithelial
cAMP: cyclic adenosine monophosphate
GLP-1R: glucagon-like peptide-1 receptor
IIH: idiopathic intracranial hypertension
i.p.: intra peritoneal
ICP: intracranial pressure
MABP: mean arterial blood pressure
RBP4: retinol binding protein 4
TBI: traumatic brain injury
